# Outcomes of a community sector model of reintegration for people with complex needs: a mixed-methods study

**DOI:** 10.1186/s40352-025-00352-6

**Published:** 2025-08-04

**Authors:** Ruth McCausland, Rebecca Reeve, Mindy Sotiri, Lucy Phelan, Vendula Belackova, Sophie Russell

**Affiliations:** 1 Present Address: UNSW Sydney, Sydney, Australia; 2 Present Address: Justice Reform Initiative, Sydney, Australia; 3 Community Restorative Centre, Sydney, Australia

**Keywords:** Prison, Post-release, Outreach, Throughcare, Reintegration, Substance use, Community sector, Program evaluation, Economic analysis

## Abstract

**Background:**

Re-incarceration rates for people leaving prison in Australia are high, particularly so for those experiencing complex needs including mental health and substance use issues, cognitive disability and homelessness. Effective models of reintegration for this group are needed to reduce incarceration and improve health and wellbeing.

**Objective:**

This article reports on the outcomes of a mixed-methods evaluation of the Community Restorative Centre’s (CRC) model of intensive casework support for people with complex needs exiting prison in New South Wales, Australia.

**Methods:**

The evaluation took a mixed-methods approach to assessing the effectiveness of CRC’s model of reintegration support which included five studies: (1) an interrupted time series analysis of 483 clients over an 11-year period to examine changes in custody and court outcomes following engagement with CRC; (2) a comparative interrupted time series analysis of 246 CRC clients vs. a de-identified comparison group of 567 individuals that did not receive CRC support; (3) a cost-benefit analysis of criminal justice vs. CRC program costs; (4) analysis of client health and well-being survey data; and (5) semi-structured interviews with 26 individuals including CRC clients (*n* = 14) and CRC staff (*n* = 12).

**Results:**

After participating in CRC programs, contact with the criminal justice system reduced significantly, including a 66% reduction in days in custody, a 63% reduction in new custody episodes, and a 62% reduction in proven offences. The comparative analysis estimated savings of $10–16 million in criminal justice costs for each annual cohort of new clients. Well-being survey data analysis indicated that clients were consistently managing drug use, self-care, relationships and resources over the time of engagement with CRC. Qualitative interviews highlighted that meeting people’s basic welfare, housing and health needs was fundamental, as was the model of support (flexible, outreach, relational and long-term) and the workers’ approach (respectful, non-judgemental, compassionate, consistent).

**Conclusions:**

An intensive, flexible, outreach model of community sector reintegration support that meets the basic welfare, housing and health needs of people with complex needs exiting prison can significantly reduce their contact with the criminal justice system and produce economic benefits for government.

## Background

The majority of people in Australian prisons have experienced multiple and intersecting disadvantage including limited access to education and employment, high rates of poverty and homelessness, cognitive disability, poor mental and physical health, and alcohol and other drug dependency (Australian Institute of Health and Welfare, 2023; Borzycki & Baldry, 2003; Kirwan et al., 2019; McCausland & Baldry, 2023). In Australia, more than one third (34.5%) of people in prison are First Nations despite making up only 3.8% of the general population, with even higher rates for First Nations women and young people and those on remand (ROGS, 2025; Australian Bureau of Statistics, 2024; Russell et al., 2022; Tubex, 2021). There are multiple structural issues that impede people who are released from prison from access to health and social services, including housing (Treloar et al., 2021). The risk of re-incarceration is particularly high for those with complex needs (Borzycki & Baldry, 2003; Doyle et al., 2022).

Throughcare and other reintegration programs are designed to assist people to transition from prison into the community, and have been shown to reduce the risk of reoffending (Fox et al., 2005; Gilbert & Elley, 2015; Schwartz & Terare, 2020). A recent systematic review of qualitative studies on throughcare suggested that the skills of the case workers and accessibility of housing and social supports are critical in achieving positive reintegration outcomes (Kendall et al., 2018). The key elements of effective reintegration support have been found to include person-centred, intensive case management (Kendall et al., 2018; Moore, 2019), approaches that are strengths-based (Angell et al., 2014; Hunter et al., 2016), provision of housing (Padgett et al., 2006; Sotiri & Russell, 2018), and ensuring continuity of care from custody to the community (Doyle et al., 2022; Edwards et al., 2022).

Models of throughcare vary across jurisdictions and can be coordinated by government services (Griffiths et al., 2017), corrections (Maycock et al., 2020), or community organisations. For people with complex needs and First Nations people, suitable models of support have been found to include localised, community-based programs, and holistic approaches to achieve trust and familiarity between workers and their clients (Day et al., 2019; Tubex, 2021). Case studies from Australia and internationally have shown reintegration programs led by the community sector in particular can make a profound difference in the lives of people who are at risk of reincarceration (Sotiri, 2020). However there remains an evidence gap regarding the efficacy of reintegration programs in particular for people with complex needs.

### Community Restorative Centre (CRC) model of support

The Community Restorative Centre (CRC) is the largest non-government organisation in NSW providing specialist support to people impacted by the criminal justice system and their families, with a particular emphasis on the provision of post-release and reintegration programs for people with complex needs. CRC operates a number of programs which aim to reduce recidivism, break entrenched cycles of criminal justice system involvement, and build pathways out of the criminal justice system and into the community (Community Restorative Centre, 2023). CRC’s clients have usually experienced significant disadvantage and have long histories of contact with the criminal justice system. CRC aims to support people who might otherwise have difficulty accessing services because of the multiplicity and complexity of their support needs. CRC is also focused on supporting people who have experienced exclusion and stigma due to their history of involvement in the criminal justice system (Churchill et al., 2017; Russell et al., 2023). While all CRC programs support people with alcohol and other drugs (AOD) issues, it also runs a dedicated AOD transitional support program (Community Restorative Centre, 2023).

CRC provides intensive, outreach case-management, which is holistic, relational, person-centred (needs-based), flexible, and long-term. While most CRC programs are funded to provide support for 12 months, some programs extend beyond that timeframe. CRC’s reintegration programs are based on a throughcare model which involves engaging with clients while they are still in prison – ideally three months prior to their release – in order to build trusting relationships and facilitate a smooth transition from prison to the community (Hamilton & Belenko, 2016; McKenna et al., 2015; Sotiri, 2016). CRC also supports people living in the community who, because of their complexity of need and experience of disadvantage, are at risk of criminalisation and imprisonment. All CRC’s programs work to support the intersecting needs of people impacted by the justice system. Staff provide practical casework support (including support with housing, health, legal issues, finances, and transport) alongside emotional and relational support. Relational case-work support involves the modelling of positive healthy and safe relationships and social connections through the interactions between workers and clients (Sotiri et al., 2021). Case-workers also offer non-judgemental, strengths-based, trauma-informed counselling.

### Studying outcomes of the CRC model of support

This study was undertaken after identification of a gap in research and evaluation regarding the efficacy of reintegration programs, in particular for people with complex needs (Sotiri et al., 2021). A mixed-methods approach was chosen to investigate the impact of participation in CRC programs on clients’ contact with the criminal justice system and to improve understanding of the mechanisms and reasons behind program effectiveness. Measuring reductions in recidivism is a common metric of the effectiveness of reintegration programs and was included as part of this study. However, an exclusive focus on recidivism has been critiqued as narrow and deficit-oriented (Edwards et al., 2022), overlooking structural factors that limit its reliability as a measure of program effectiveness (Sered, 2021). Other definitions of ‘success’ in reintegration of people after release from prison have increasingly been included in research and evaluation, including qualitative investigations of the perspectives of people with lived experience of the criminal justice system and the role of systemic issues (Andersen et al., 2020; Johns, 2015; Maruna, 2020). Taking a mixed methods approach enabled analysis of the impact of CRC’s programs on clients’ recidivism rates as well as on their health, wellbeing and associated social and economic outcomes, informed by the expertise and voices of people who have experienced reintegration and those working intensively with them.

The study sought to answer the following questions on the impact of participation in CRC’s programs:

1. What is the effectiveness, including cost-effectiveness, of CRC’s model of support on clients’ court and custody outcomes?
2. What are the health and social outcomes of CRC’s model of support?
3. What are the factors that contribute to the effectiveness of CRC’s model of support?

## Methods

The study included five sub-studies. Three focused on court and custody outcomes and associated costs and benefits: (1) an interrupted time series analysis of the court and custody outcomes of 483 CRC clients over an 11-year period pre and post commencement with CRC; (2) a comparative interrupted time series analysis of the court and custody outcomes of 246 CRC clients who were referred to CRC from custody vs. a similar cohort of people who exited custody in the same period but did not receive CRC support; (3) cost-benefit analysis comparing the estimated cost savings to the justice system of CRC participation (based on comparative interrupted time-series analysis of criminal justice costs) with the cost of CRC programs. Two further studies assessed clients’ health and social outcomes: (4) analysis of Personal Wellbeing Index survey data; and (5) semi-structured interviews with CRC clients (*n* = 14) and CRC staff (*n* = 12).^1^ The semi-structured interviews also explored the mechanisms of CRC’s model of support. While both the qualitative and quantitative components of the study addressed similar research questions, they involved distinct samples and were designed to provide complementary perspectives rather than convergent findings, consistent with a multi-method mixed-methods approach. Each sub-study method is detailed below.

### Study 1: analysis of court and custody outcomes of CRC clients

Study 1 comprised an interrupted time series analysis of criminal justice system outcomes of CRC clients. This study included 483 CRC clients who participated in transition and AOD programs between 2014 and 2017, whose client records were linked by the Bureau of Crime Statistics and the Research (BOCSAR) to the NSW Reoffending Database (ROD) and deidentified. Approximately 70% (337 of 483) people in the study sample were referred to CRC while in custody and the remaining 30% were referred from the community. Reoffending outcomes were assessed for clients referred from the community and those referred from prison separately as well as for all CRC clients together. Limiting the time frame for the sample selection to 2014–2017 was to allow for a minimum 2-year follow-up period, and because CRC programs had settled into a more consistent format of service delivery due to significant reforms in funding models in 2012/2013.

Data from the ROD were used to analyse clients’ court and custody outcomes. The ROD contains data on finalised legal actions within the NSW criminal justice system. The variables used were number of finalised court appearances, proven offences, new custody episodes, and days in custody. Court outcomes were counted based on offence dates. The date of the principal offence was used for finalised court appearances, and the individual offence date for proven offences. If the offence date was missing this was imputed using the charge date and, if that was also missing, first appearance date. Where a custody episode had no end date the person was assumed to still be in custody at the end of the observation period. If a person was already in prison and then commenced a new sentence, any overlapping days in custody were only counted once.

Each person’s data were shaped into a panel representing 11 annual time periods from January 2009 to December 2019. The index year was split into two periods, the time before and the time after engagement with CRC support in the community. For each period, the models were adjusted for the number of days available where the year was not fully observed.^2^ Random effects models were used, which take into consideration the panel nature of the data (repeated observations for the same individuals over time).

The interrupted time-series models examined time-trend in court and custody outcomes before each client engaged with CRC, any shift after engagement commenced, and any change in trend after engagement with CRC. The models included multiple time points to control for underlying trends and for differences between groups (Lopez Bernal et al., 2018), and controlled for participant characteristics which might influence their risk of reoffending, in particular: gender, Aboriginal status, age, having history of juvenile incarceration, and having contact with CRC prior to 2014 (this was to control for clients who had engaged with CRC before 2014 and thus may have experienced different trajectories). In addition, to help control for regression to the mean (i.e. a natural decline in justice contacts immediately following an episode in custody), the analyses were adjusted for outcomes during the period leading up to the intervention.

### Study 2: comparative interrupted time-series analysis of court and custody outcomes

To help to control for any changes over time that would still have occurred in the absence of CRC support, a comparison study was also undertaken. Permission was granted to draw on a linked administrative databank (the UNSW Mental Health Disorders and Cognitive Disability in the Criminal Justice System (MHDCD) Databank) of a cohort of people who have been in prison in NSW. While CRC clients referred from prison received the same model of support as those referred from the community, it wasn’t feasible to find a comparison group for clients referred while in the community. Therefore, the ‘intervention group’ was restricted to clients referred to CRC from prison, which also allowed for a comparable index date across the two groups.^3^ For the comparative analysis, the ‘intervention group’ consisted of 246 CRC clients who were referred directly from prison and who had their first core program participation in 2014 to 2017. The ‘comparison group’ consisted of 567 people from the MHDCD Databank who had an AOD diagnosis, at least one prison exit during the period 2014 to 2017 and who had never been a CRC client.

Descriptive statistics of the CRC cohort were extracted from the CRC internal database and linked ROD data; for the comparison group, these were taken from the MHDCD databank. CRC clients’ mental health and cognitive disabilities were not always recorded within a client’s records. Therefore, if a client was identified with a condition in any of their CRC records they were assumed to have that condition. Demographics for CRC clients were from the ROD data where available and completed from CRC data otherwise. Aboriginality is known to be unreliably captured in administrative data and was recorded inconsistently in the BOCSAR ROD and CRC client databases. Therefore, if a CRC client was ever recorded as being Aboriginal, in either the CRC data or in the BOCSAR data, they were counted as Aboriginal. Diagnoses and demographic variables in the MHDCD dataset were compiled previously from a range of linked datasets (Baldry, E. Dowse, L. and Clarence, M., 2012).

Table 1 summarises characteristics of the intervention group, the comparison group, and CRC clients overall. All three groups were similar in terms of their age (mean was 37 years for CRC clients and 38 years for the comparison group). CRC clients were more likely to be female (many of CRC’s programs prioritise women as a specific risk group). CRC clients were also more likely to be First Nations and less likely to have had a youth justice custody episode than the comparison group (the latter may be related to the above-mentioned gender difference). CRC clients were more likely to have a dual diagnosis (AOD and either cognitive disability or mental health diagnosis) than the comparison group, who were more likely to have received all three diagnoses. To account for the differences, these characteristics were controlled for in all analyses. Models with interactions were also run to evaluate whether the impact of CRC support differed for male and female clients. Women had lower average rates of offending across all outcomes measured. However, the models with interactions did not show any statistically significant differences in the change in outcomes by gender before and after the index date, nor differences by gender between CRC clients and the comparison group. Therefore, results without interactions are presented in this paper.

**Table 1:** Study sample characteristics - CRC clients, CRC clients referred from custody, and comparison group

Characteristic	All CRC clients (*N* = 483)	CRC intervention group (*N* = 246)	Comparison group (*N* = 567)
Age at index date (range)	37.2 (19.0 to 69.5)	37.7 (24.3 to 59.2)	38.0 (24.1 to 60.0)
Male	45.6%	42.3%	93.1%
Aboriginal	48.2%	46.3%	36.7%
Any juvenile custody	16.6%	16.7%	48.7%
AOD only	17.8%	18.7%	11.6%
AOD and CD or MH	63.2%	65.5%	48.3%
AOD and CD and MH	19.1%	15.9%	40.0%
First engaged in prison	69.8%	100.0%	100.0%
First engaged in community	30.2%	0.0%	0.0%
Participated in CRC programs before 2014	8.3%	0.0%	0.0%

AOD – alcohol and other drugs; CD – cognitive disability; MH – mental health

Comparative interrupted time series modelling was used to estimate differences between the intervention group and a comparison group before and after the intervention (the index date), including multiple time points to control for differences in underlying trends between the groups (Jacob et al., 2016; Lopez Bernal et al., 2018). Unlike simple difference in difference analysis which requires baseline trends to be the same, comparative interrupted time series analysis enables any differences in underlying trends to be tested and controlled for (Lopez Berna et al. 2019). The models also control for other observed differences which may be associated with criminal justice outcomes. However, some residual confounding due to unobserved differences may remain and is discussed in the results section. The comparison analysis was conducted for a shorter time-span than the interrupted-time series analysis because data were limited for the comparison group; see details in the next section.

### Study 3: cost-effectiveness assessment based on comparative interrupted time-series analysis of criminal justice cost and on estimated cost of CRC programs

A comparative interrupted time series model was used to compare the predicted difference in criminal justice costs over time for the intervention group compared to the comparison group, controlling for other differences and underlying trends. Each analysis was restricted to the duration of the relevant data in the databank, which for custody outcomes was up to 2018 and for court outcomes up to 2016.^4^

The cost of court appearances and custody episodes (per person per day in prison) were taken from the Australian Productivity Commission’s Report on Government Services (ROGS, 2020). The estimated social cost of crime and the cost of police interactions for each proven offence was calculated drawing on a study by Smith and colleagues (2014). As data on police interactions for CRC clients was not available, police costs were calculated assuming 6.5 police interactions per finalised court appearance (the average number of police interactions per finalised court appearance among the MHDCD cohort which was applied equally to both groups).

The unit costs described above were summed per person for each of the years when all of outcome data were available (2009 to 2016) to give the total criminal justice cost per person per annum. An overall cost model was then estimated, indicating the savings to the criminal justice system for clients who received CRC support relative to the comparison group. Results from the costs model were used together with the average cost of delivering CRC programs per person, to estimate the net benefit of participation for an annual intake of 275 new CRC clients, over three years post commencement with CRC.

### Study 4: client survey data on wellbeing

To assess CRC client wellbeing over one year of receiving CRC support, 233 client Substance Use Recovery Evaluator (SURE) surveys were completed by 147 CRC AOD program clients. The AOD program offers case management like other CRC’s programs but provides also additional AOD counselling. SURE is a validated tool intended for people in recovery from substance use and is routinely collected from CRC clients for operational and funding purposes (Neale et al., 2016). It consists of 26 questions in five key areas: substance use, self-care, relationships, material resources, and outlook on life. It is designed as a holistic health and wellbeing measure and relies on self-reported information, allowing for the tracking of changes over time. Mean scores in each of the five areas per client were calculated. Next, distribution of the mean scores per month was assessed. Months were calculated according to the timespan between starting the CRC program and filling in the SURE.

In this analysis, we tested for the difference between the average score per area in each month, compared to month 1, to assess changes in clients’ health and wellbeing over time. T-test comparing values with month 1 was performed for each of the five SURE areas as well as for individual questions.

The reliance on secondary data (surveys administered by caseworkers in the course of their casework duties) resulted in fewer surveys than anticipated and varying numbers of completed surveys in any given time period. The variability in survey completion appeared to be related to some caseworkers finding administration of the survey challenging or not a priority rather than an indicator of client wellbeing; it isn’t possible to conclude whether or how those who completed the survey were similar or different to other clients. The inconsistency of survey administration meant that we were not able to track individuals’ changes over time (as initially planned), but instead focused on average shifts in health and wellbeing for the whole cohort over a 12-month period. As a result, the analysis presented reflects group-level trends across the cohort rather than changes for individual participants as that could not be reliably assessed.

### Study 5: semi-structured interviews with clients and staff

 The qualitative study was comprised of 26 qualitative in-depth face-to-face interviews with CRC clients (*n* = 14) and staff (*n* = 12), numbers which are considered sufficient for theoretical saturation in qualitative research (Creswell, 2014; Crouch & McKenzie, 2006). Of the 14 clients that were interviewed, half (*n* = 7) identified as male and the other half as female. Clients were aged between 30 and 60 years, with an average age of 43. Half (*n* = 7) of the clients were First Nations people. Of the CRC staff that were interviewed, four were Managers and eight were Caseworkers. Two of the staff also had lived experience of incarceration. CRC clients were recruited through the CRC Research Officer. Clients were given a $30 gift voucher as reimbursement for their time. Staff were not provided with any reimbursement for their participation.

The interviews followed a semi-structured interview style using a discussion guide. The client discussion guide covered their life history, background and experience as a client of CRC, and the staff discussion guide covered roles and responsibilities at CRC, clients’ needs and how to meet these, and their perception of the efficacy of CRC support and how it could be improved. The interviews took approximately 60 min and were conducted either at the CRC office or another suitable location. Braun and Clarke’s (2006) six stages of thematic analysis were followed: familiarisation of data; generation of initial codes; combining codes into themes; reviewing themes; determining significance of themes; and writing up.

### Ethical considerations

The study received ethics approval from University of New South Wales (UNSW) Human Research Ethics Committee (HREC) (HC190209 and HC190724) and from the Aboriginal Health and Medical Research Council (AH&MRC) HREC (1575/19 and 1682/20). The Corrective Services NSW (CSNSW) Commissioner provided a letter of approval (D20/0316317) in relation to including custody data in the ROD data provided for this study (BOCSAR ref. rod2019080). An Aboriginal Reference Group (ARG) was formed and consulted on in the process of this study in 2020 and 2021. The ARG was comprised of three Aboriginal staff members of CRC who had not been involved in the programs being evaluated, and one external consultant.

For the qualitative study, interviewees read (or had read aloud to them if requested) and then signed the Participant Information Sheet and Consent Form before their interview. Interviewees were advised that their participation was optional, and their choice to participate would not negatively impact them continuing to receive support or employment (in case of staff) from CRC. Participants were asked if they consented to the interview being audio recorded. All participants were advised that the interview would be confidential and de-identified in any reporting. The interviewer advised the participants that they could end the interview at any time should they feel uncomfortable with the questions or process. Aboriginal participants were given the option of their interview being conducted by a First Nations researcher. However, this option was not taken up.

## Results

The following sections outline the results for each of the sub-studies. The first three focus on results from quantitative analysis related to court and custody outcomes and program cost-effectiveness, and the final two studies focus on health and social outcomes related to survey findings and qualitative interviews, as well mechanisms of effectiveness in CRC’s programs.

### Study 1: interrupted time-series of court and custody outcomes for CRC clients referred from prison and the community

Prior to engaging with CRC, the number of finalised court appearances for CRC clients was increasing by 15.6% (IRR 1.156) year on year. The number of proven offences was increasing by 20.1% (IRR 1.201), new custody episodes by 25.1% (IRR 1.251), and the number of days in custody by 21.3% (IRR 1.213) every year. After commencement with CRC, the number of clients’ finalised court appearances fell by 47.8% (1-0.522) for CRC clients overall. Proven offences, new custody episodes, and the number of days in custody decreased by 62.1% (1-0.379), 62.6% (1-0.374), and 65.8% (1-0.342) respectively. After engaging with CRC, the trend over time also fell by 17.3% (1-0.827) for finalised court appearances, by 17.9% (1-0.821) for number of proven offences, by 21.6% (1-0.784) for new custody episodes, and by 19.3% (1-0.807) for days in custody, see Table 2.

**Table 2:** Interrupted time-series analysis of criminal justice outcomes for CRC clients

	IRR	Std. Err.	z	*p*> *z*	[95% Conf. Interval]	
**Number of finalised court appearances**						
Initial time trend pre-CRC commencement	1.156	0.013	12.86	0.000	1.131	1.182
Shift post-CRC commencement	0.522	0.043	-7.85	0.000	0.444	0.614
Trend change post-CRC commencement	0.827	0.020	-7.78	0.000	0.789	0.868
**Number of proven offences**						
Initial time trend pre-CRC commencement	1.201	0.014	15.52	0.000	1.174	1.229
Shift post CRC commencement	0.379	0.033	-11.00	0.000	0.319	0.451
Trend change post CRC commencement	0.821	0.021	-7.84	0.000	0.782	0.863
**Number of new custody episodes**						
Initial time trend pre-CRC commencement	1.251	0.016	17.74	0.000	1.220	1.282
Shift post-CRC commencement	0.374	0.034	-10.70	0.000	0.312	0.448
Trend change post-CRC commencement	0.784	0.022	-8.84	0.000	0.743	0.828
**Days in custody**						
Initial time trend pre-CRC commencement	1.213	0.012	19.04	0.000	1.189	1.237
Shift post-CRC commencement	0.342	0.029	-12.82	0.000	0.290	0.403
Trend change post-CRC commencement	0.807	0.020	-8.84	0.000	0.770	0.847

*Notes*: Analyses were adjusted for the following covariates: commencement with CRC pre-2014, history of juvenile custody, gender, age at index date, Aboriginality, and intervention period

**Fig. 1:**
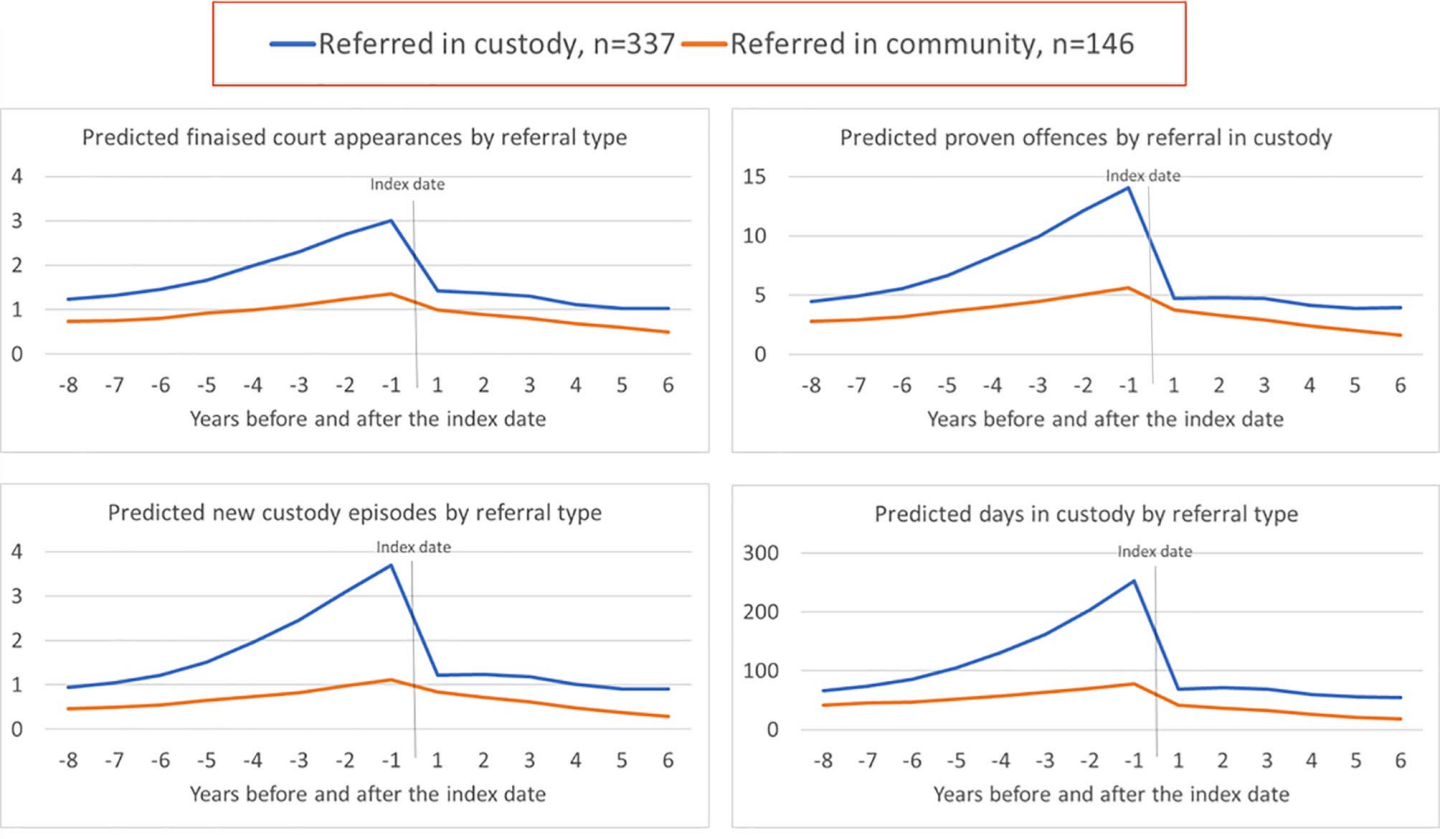
Predicted criminal justice outcomes for CRC clients referred from the community and custody

Models with interactions were also run to explore whether outcomes differed for clients referred to CRC from prison compared to those who were referred from the community. At the start of the observation period (2009), people who were referred to CRC from prison began with 57.8% more finalised court appearances, 45.9% more proven offences, 83.1% more custody episodes, and 43.3% more days in custody than people who were referred from the community, and these differences were initially increasing over time. Following CRC support, clients who were referred from prison had a greater decrease in all re-offending outcomes than clients who were referred from the community. After CRC intervention, both groups had a similar trend in court and custody outcomes alike, with clients referred from prison maintaining a slightly higher rate of court and custody episodes than those referred from the community (see Fig. 1).

### Study 2: comparative interrupted time-series analysis of court and custody outcomes for CRC clients and comparison group

The comparative interrupted time series analysis included only those CRC clients who were referred from custody. At the start of the observation period, there was no significant difference in the number of finalised court appearances, the number of custody episodes, or the number of days in custody between CRC clients referred from custody (‘intervention group’) and the ‘comparison group’. However, CRC clients referred from custody had 18.4% (1-0.816) fewer proven offences than the comparison group at the start of the observation period.

**Fig. 2:**
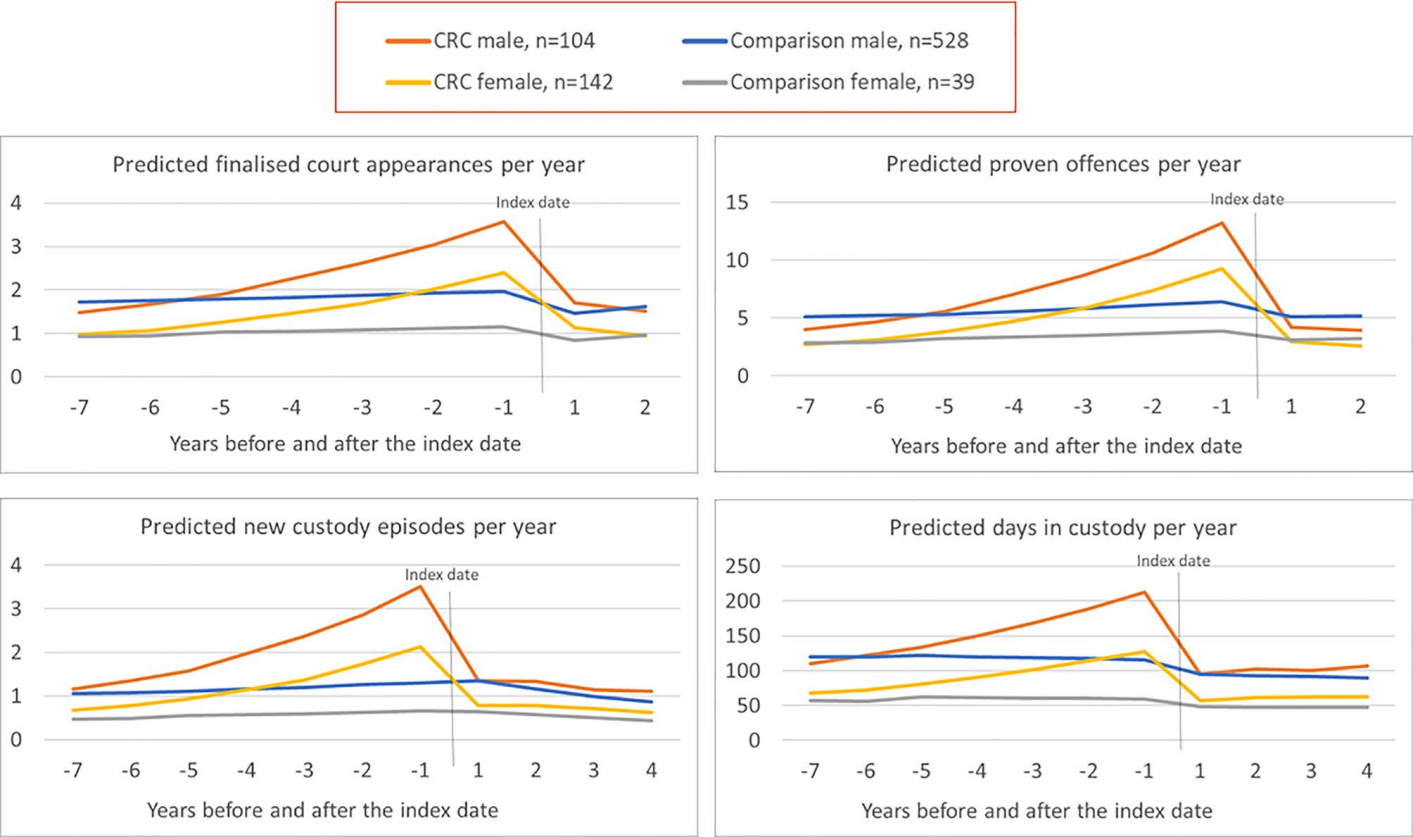
Predicted criminal justice outcomes for CRC clients and comparison group

Baseline trends in criminal justice outcomes over time were higher for CRC clients referred from prison than among the ‘comparison group’. The differences suggest that CRC targeted clients who were, initially, at a greater risk of repeated contact with the criminal justice system. The differences in baseline trends and observed characteristics were controlled for in the models; however, residual confounding from unobserved differences may remain. For example, as CRC clients have more complex needs than the comparison group, they may be less likely to receive/benefit from mainstream services in the absence of CRC support. Therefore, the results of the comparative interrupted time series models may underestimate the program effects.

The results are presented in Fig. 2 as graphs of predicted outcomes from the models. The results are shown separately for clients who identified as males and females due to the different gender ratios between the CRC cohort and comparison group.

For people who received CRC support (‘intervention group’), there was a decrease in court appearances after the index date of 43.8% and a decrease in the trend over time of 20.3%. Together, these decreases were significantly higher than for the comparison group, for whom court appearances decreased by 36.1% after the index date with no significant decrease in trend over time.^5^

Also, following index date, there was an immediate decrease in the number of proven offences for CRC clients (62.8%) which was much higher than for the comparison group (25.5%), and there was no significant change in the trend over time for either group. For the number of custody episodes, CRC clients had a significant downward shift of 63.3% after the index date and there was no evidence of a downward shift for the comparison group. For both groups, after the index date, there was a decrease in the trend over time in custody episodes of 17%. Finally, for the number of days in custody, after index date, CRC clients saw a decrease of 58.8% while the comparison group decreased by 16.3%. Following the initial decrease there was no change in the trend over time in custody days for the comparison group or for the group who received CRC support, see Fig. 2.

Overall, women had 38.1% fewer finalised court appearances than men. They also experienced fewer proven offences (35.7%), fewer custodial episodes (45.2%), and fewer days in custody (43.8%) than men. Models including gender interactions found that this gender difference did not change significantly over time and that it did not differ for the CRC ‘intervention group’ relative to the ‘comparison group’.

### Study 3: cost-effectiveness assessment based on comparative interrupted time-series analysis of criminal justice cost and on estimated cost of CRC programs

There was no significant difference in initial criminal justice costs of CRC clients and the comparison group at the start of the observation period. However, consistent with the previous models, the CRC group had a larger increase in cost trend of $4,215.02 per year prior to commencing CRC programs. This difference in the baseline trend was controlled for in the model.

 After the index date, there was an initial decrease in criminal justice costs of $26,874.35 and an increase in trend of $6,159.31 for the comparison group. For people who received CRC support the initial decrease was $30,490.16 (26,874.35 + 3615.81) and the trend in costs decreased by $3,492.98 per year (9652.28-6159.31). A joint significance test showed that the differences in shift and trend for the CRC group relative to the comparison were statistically significant ( *p*  = 0.000). Overall, being a CRC client relative to being in the comparison group was associated with criminal justice cost savings of $13,268.09 (3615.81 + 9652.28) one year after the intervention which increased by $9,652.28 per year for each additional year after the intervention (see Table  3 ).

**Table 3:** Comparative interrupted time-series analysis of criminal justice costs for CRC clients and a comparison group

Criminal justice costs	Coef.	Std. Err.	z	*p* > z	[95% Conf. Interval]	
Female (cf. male)	-17,759.22	2,486.07	-7.14	0.000	-22,631.83	-12,886.62
Initial difference in CRC group (cf. comparison group)	479.28	3,647.64	0.13	0.895	-6,669.96	7,628.52
Comparison group time trend	1,151.45	370.24	3.11	0.002	425.80	1,877.11
Difference in CRC time trend (cf. comparison group)	4,215.02	578.57	7.29	0.000	3,081.04	5,348.99
Comparison group shift post index date	-26,874.35	5,265.85	-5.10	0.000	-37,195.23	-16,553.47
Difference in CRC shift post index date (cf. comparison group)	-3,615.81	13,360.85	-0.27	0.787	-29,802.58	22,570.97
Comparison group trend change post index date	6,159.31	2,177.16	2.83	0.005	1,892.16	1,0426.45
Difference in CRC trend change post index date (cf. comparison group)	-9,652.28	6,728.67	-1.43	0.151	-22,840.24	3,535.68

*Notes*: Also adjusted for age, Indigenous status, complexity, any juvenile custody, and the period the intervention commenced. Costs have been annualised based on the number of days in the period. Sample size 7,144 observations for 813 individuals. The average number of observation periods per person is 9 (range from 6 to 9). Coefficients are presented in Australian dollars (2019)

 The average cost of delivering CRC programs, was $7,992.35 per female client and $14,797.62 per male client. Based on average time in the program (450 days) approximately 80% of the cost of CRC participation was incurred in the first year and 20% in the second year post-commencement with CRC. To calculate the total cost-effectiveness of CRC’s programs, the cost of program delivery was compared to the estimated cumulative cost savings from reduced contact with the justice system over three years ($68,761.12 per client). This gave a net benefit of $60,768.77 per female client and $53,963.50 per male client. For a typical annual intake of 275 clients (63.1% of whom were female and 36.9% male) the estimated net benefit of the program over 3 years was $16 million (see Table 4).

However, a more conservative estimate may be made, considering that the comparative interrupted time-series models were based on the 70% of clients who were referred from custody. Even though community- based clients also benefited from the program according to the time-series analysis, the lack of comparison group prevented the economic analysis being completed for them. Therefore, it may be assumed that the savings would only apply to 70% of CRC clients (i.e. those referred to CRC from custody), reducing the per-person savings to $48,132.79 (68,761.12 × 70%), on average. The average net benefit of CRC programs would then be $40,140.43 per a female and $33,335.16 per a male client, for a total net benefit of $10,347,883.99 for an intake of 275 clients. This is a conservative estimate because it assumes there were no cost savings to people referred to CRC from the community.

**Table 4:** Cost of criminal justice system for CRC clients and comparison group; cost-effectiveness of CRC program for 275 clients

	Year 1 post index date	Year 2 post index date	Year 3 post index date	Estimated total	Conservative estimate**
**Criminal justice cost ($)**					
Change comparison group (-26,874.35 + 6,159.31 x years)	-20,715	-14,556	-8,396	-	-
Change CRC group (-30,490.16 -3,492.98 x years)	-33,983	-37,476	-40,969	-	-
Cost savings per CRC client relative to comparison group	13,268	22,920	32,573	68,761	48,133
**Cost-effectiveness analysis ($)**					
Cost of CRC per female client	6,394	1,598	-	7,992	7,992
Cost of CRC per male client	11,838	2,960	-	14,798	14,798
Net benefit per female client (savings less costc)	6,874	21,322	32,573	60,769	40,140
Net benefit per male client (savings less cost)	1,430	19,961	32,573	53,964	33,335
**Total net benefit of 275 new clients per year** ^*^	$1,337,819	$5,725,376	$8,957,481	$16,020,677	$10,347,884

* Of the 275 new clients, 63.1% are female and 36.9% are male (a small number of clients of unspecified gender were allocated evenly between male and female for the purpose of this analysis)

** The conservative estimate assumes that savings may only apply to 70% of CRC clients (i.e. those referred to CRC from custody), even though both community and custodial clients outcomes improved according to the interrupted time-series analysis

### Study 4: client survey data

**Fig. 3:**
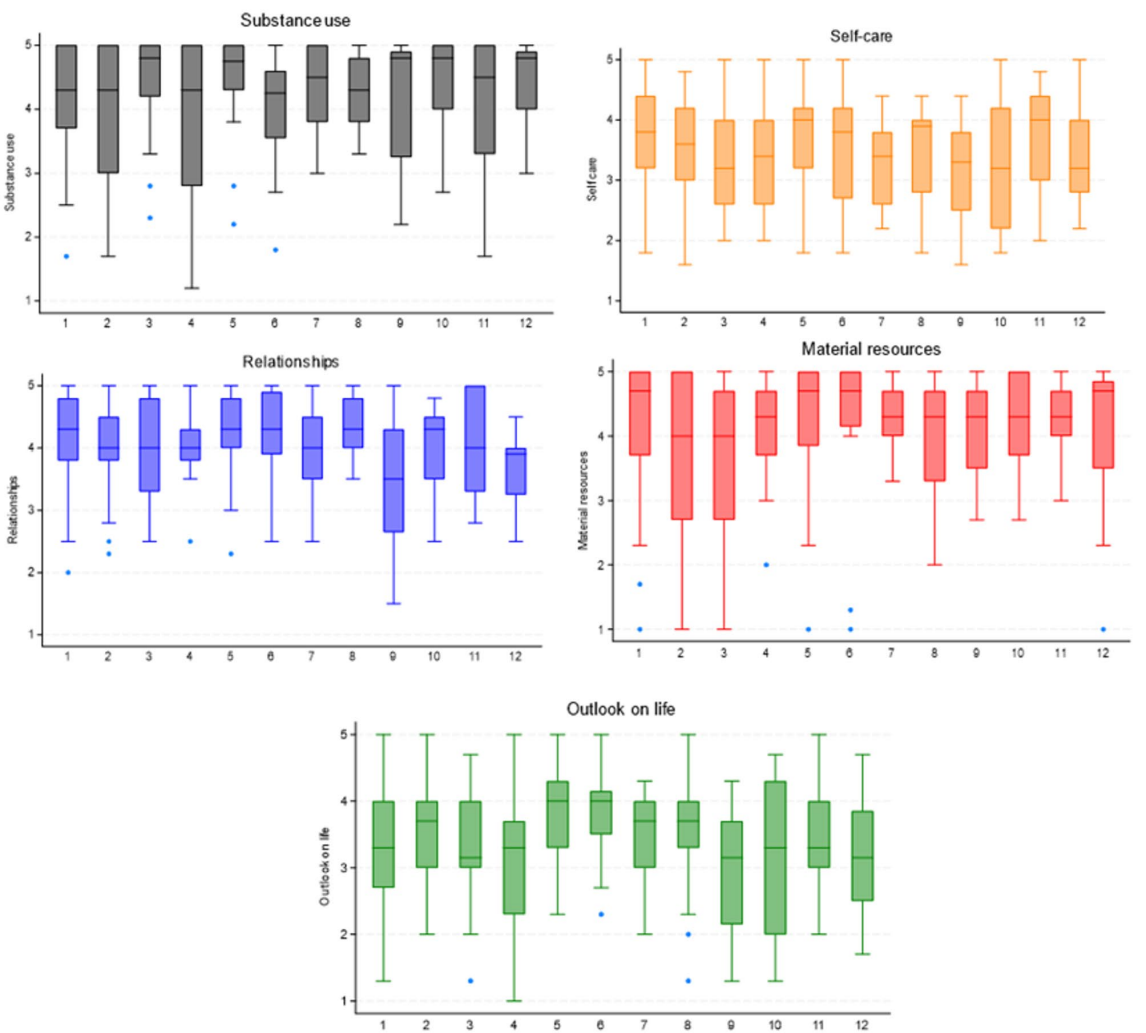
Distribution of wellbeing outcomes according to SURE assessment.

The purpose of this analysis was to assess CRC clients’ wellbeing over the course of 12 months of being a CRC client by calculating the average value per each of the five SURE domains. The following scores were indicated on a scale 1 to 5 where one was the worst outcome (e.g. “I have coped with problems without misusing drugs or alcohol” - none of the time), five the best outcome (“all of the time”), and two to four were assigned to the values in between (a little or a fair amount of time and most of the time); all questions were coded in a way that the best outcome yields the highest value. The distribution of the values per domain for each of the 12 months is shown in Fig. 3.

In summary, substance use scores indicated there was a consistent (but relatively low) level of use throughout the period and relatively high scores in terms of various forms of coping (managing pain, spending time on hobbies unrelated to substance use). Self-care scores also remained fairly consistent over time and reflected a reasonable level of reported self-care, although there were less consistent and lower scores in the domains in terms of sleeping well and looking after physical health, and greater fluctuations in terms of eating well. There was consistency over time in terms of how people viewed their relationships, although this was rated much more highly in terms of treating others with respect when compared to the experience of being treated with respect or feeling supported by others. Material resources responses were also consistent over time, with scores for managing money lower than stable housing and regularity of income. When comparing the scores each month with month 1, most indicators were stable (didn’t change). Only a few statistically significant shifts were observed. The few statistically significant changes involved improvements in the mean values at month 5 for substance use (t= -1.8607, Pr(T < t) = 0.0339) and outlook on life (t= -2.6009, Pr(T < t) = 0.0059). For outlook on life, this positive change persisted through month 6 as well. Looking at the particular SURE questions which contributed to the significant changes, these were:

“I have been spending my free time on hobbies and interests that do not involve drugs or alcohol”, “I have felt happy with my overall quality of life”, “I have felt positive”, and “I have had realistic hopes and goals for myself”. There was also a significant improvement in the question “I have drunk too much” in month 12 when all of the clients responded “never”, even though this did not constitute a statistically significant change in the substance use area in month 12.

On the other hand, there were deteriorations in self-care as well as relationships in month 9 of CRC support (t = 1.9310, Pr(T > t) = 0.0298 and t = 2.2700, Pr(T > t) = 0.0139 respectively). Correspondingly, there were significant deteriorations in month 9 (compared to month 1) in the following questions: “I have been taking care of my physical health”, “I have been eating a good diet”, and “I have been treated with respect and consideration by people around me”.

Boxplot description: The center line in each box represents the 50th percentile (median) of values in each SURE domain by month of being a CRC client. The bottom of each box represents the 25th percentile of the SURE domain score and the top of each box represents the 75th percentile. Sample size: month 1 *n *= 33, month 2 *n *= 34, month 3 *n *= 22, month 4 *n *= 19, month 5 *n *= 28, month 6 *n *= 16, month 7 *n *= 13, month 8 *n *= 14, month 9 *n *= 16, month 10 *n *= 11, month 11 *n *= 11, month 12 *n *= 16.

### Study 5: qualitative data from CRC clients and caseworkers

The interviews with CRC clients and staff revealed important insights into the social and demographic background of CRC clients and their histories of contact with the criminal justice system, and how CRC’s model works to support clients. The following sections detail qualitative findings and highlight key themes from the interviews, organised into two categories: first, the histories, backgrounds and criminal justice system experiences of CRC clients, and second, how CRC supports them to break cycles of substance use, disadvantage and criminal justice system contact.

#### Intersecting and compounding disadvantage

CRC clients who participated in this study described facing multiple, intersecting and compounding forms of disadvantage. Central to their post-release experiences is the intersection of socio-economic marginalisation with health and psychosocial disadvantage, including substance use. The severity of disadvantage reported by participants had a profound impact on all aspects of their lives and ultimately their entanglement in the criminal justice system. Importantly, imprisonment itself constituted a distinct and self-perpetuating form of disadvantage, reinforcing social exclusion and economic instability. Understanding this incarceration disadvantage is critical in supporting people to overcome the complex challenges of navigating life after prison, as well as addressing the structural barriers to reintegration. Despite the intersecting and compounding disadvantages encountered by CRC clients, study participants repeatedly displayed an extraordinary ability to survive adversity and a resilience and adaptability in response to trauma from which much can be learned.

##### Socio-economic marginalisation

CRC clients described lifetimes of socio-economic marginalisation, including homelessness, poverty, low levels of formal education, unemployment or precarious employment, unmet disability needs, chronic poor health, mental illness, problematic substance use, violence and abuse, and repeated imprisonment. As one CRC staff member explained:

The majority of people we work with will essentially be homeless on release. A lot of people have mental health issues, cognitive disabilities, low literacy, not much in the way of employment, education, training… just come from very entrenched backgrounds of disadvantage. (CRC Staff 1)

Many of these socio-economic disadvantages were noted to be intergenerational and compounding over time. Interviews with First Nations respondents revealed that their experiences of disadvantage were shaped by the ongoing impacts of colonisation, intergenerational trauma, and systemic racism.

##### Health, trauma and substance use needs

CRC clients reported long-standing histories of trauma, often stemming from complex and unstable childhoods. Many experienced neglect, physical and sexual abuse, and early exposure to substance use:

Because of my history, I have found it really, really hard to trust anyone. Being adopted and my own adoptive parents abusing me as a child… it’s been a really hard life. (CRC Client 3)

I’ve been sexually abused as a child. I’ve been living through 24 years of domestic violence. (CRC Client 4)

Some had their first interactions with the criminal justice system at a young age after being removed from their families and placed into out-of-home-care, creating a pattern of dislocation and institutionalisation. For many, these early traumas carried into adulthood, with participants recounting experiences of violence and other forms of interpersonal and institutional abuse. Many female participants in particular described cycles of intimate partner violence and its impact on their substance use:

I used [substances] on and off when I was a teenager. And then I had my first son when I was 18 and I gave up… I was in a domestic violence relationship… I started taking drugs again to get my mind off everything… but it just made it worse. (CRC Client 11)

[Ex-partner] said he’s gonna take a hit and kill me… because of my PTSD and the trauma that I suffered during childhood and my domestic violence keeps taking me back to an addiction. (CRC Client 3)

The interviews highlighted the cyclical nature of trauma, substance use, and mental illness, which for some, was diagnosed for the first time in prison. Substance use was frequently described as a coping mechanism for managing trauma and life experiences, and many described it as closely linked with their criminalisation and imprisonment:

I’ve been on drugs my whole adult life…and most of my childhood… the drugs were just to mask any problems I had. And as the problems would get worse, I’d just choose heavier drugs. (CRC Client 12)

Alongside intersections of substance use and mental illness, staff highlighted the prevalence of cognitive disabilities and acquired brain injuries, conditions which significantly impact CRC clients’ ability to navigate the criminal justice system and life in the community. Clients also described living with various physical health issues and chronic conditions, including mobility issues, dental problems, cancer, diabetes, epilepsy, and Hepatitis C, further compounding their disadvantage.

##### Incarceration disadvantage

The experience of imprisonment emerged as a distinct and perpetuating form of disadvantage. Most participants described long histories of contact with the criminal justice system, often beginning early in life and continuing throughout adulthood. One participant recounted:

I’ve got something like 71 convictions… first lot of sentences were like six months, six months. I ticked three of those in a row. The next one was 18 months… seven years… got 6 1/2 years… 18 months. (CRC Client 6)

Participants also highlighted the impact of incarceration on their families, many of whom had also been imprisoned. While imprisonment was normalised amongst some clients, it remained a deeply damaging experience, shaped by experiences of violence and abuse, and often magnifying pre-existing traumas:

I witnessed a couple of deaths in jail… very violent, very violent, violent situations. And that really scared me, and it just really just shocked me. (CRC Client 1)

Post-release, participants faced ongoing exclusion from employment, housing, and other services (such as residential rehabilitation) on the basis of their criminal records, reinforcing their marginalisation:

Usually [when you get out of prison] you’ve got nowhere to live. You gotta do all these things and you’ve got nowhere to live [but] here on the street. You got no money it’s just… really hard [and] you can’t get a job anywhere because you got a criminal [record]. (CRC Client 9)

The backdrop of disadvantage for CRC clients was threaded throughout the interviews and understood by both clients and staff as critical to informing the nature of the support provided by CRC, and in understanding its effectiveness.

#### Breaking cycles of disadvantage: the role of CRC support

Interviews with clients highlighted two key components of the CRC service model: the practical support provided and the relational approach. The interviews with clients identified the success of CRC’s model lies not only in the personal tailoring of practical support based on client needs, but also in the method of delivery and the relational, person-centred, non-judgemental, and flexible approach.

##### Practical and legal support

Participants described the necessity of accessing basic services to rebuild their lives post-release, including housing, healthcare, legal advice, and day-to-day assistance.

Securing stable housing was identified as the most urgent need. Staff emphasised housing as foundational to stability and reintegration: ‘unless you have somewhere safe, secure, long-term to live… nothing else really works. Life’s too unstable, unpredictable’ (CRC Staff 2). Staff explained that homelessness is associated with heightened risks for clients, such as increased exposure to substance use, violence and interactions with police, all of which increase the risk of returning to prison. This was echoed by clients:

If I didn’t have the accommodation, I would have had to go to a halfway house… and I just have this feeling that I would’ve got in trouble again… for me, that was the number one thing and talking to boys in jail – they’re all homeless. If they had that accommodation it’d just change everything. (CRC Client 1)

CRC support extended beyond securing housing to include furnishing homes and providing essentials through brokerage schemes:

They helped me get stuff and move whatever furniture I had there… I needed a washing machine. I needed a fridge. I needed a bed… they linked me to the right services to get that stuff. (CRC Client 11)

Many CRC clients left prison without financial resources or bank accounts. CRC assisted with setting up bank accounts, applying for Centrelink (welfare), and managing budgets. Workers often accompanied clients to appointments and advocated on their behalf:

[CRC caseworker] picks me up… he makes sure I get to [my appointments]. If I didn’t get to those appointments I wouldn’t get the NDIS or Disability Support Pension… He did all the paperwork for that cos I would never been able to do it all. (CRC Client 1)

Clients also described the benefits of learning financial budgeting skills:

I learned how to shop and have a budget. Put so much away for my food, my rent was automatically taken out, I had my electricity taken out, so I didn’t have to worry about those… Now I just put money away for my food and then I have money in the bank…. (CRC Client 6)

Legal assistance was equally critical. CRC workers assisted with court advocacy, meeting parole requirements and obligations, and child custody issues. Clients valued this support in helping them to successfully meet their legal requirements:

I don’t think I would have finished my parole…I was very lucky to have [CRC worker] take me to all my parole appointments. (CRC Client 2)

Coming into the parole office, talking to the parole officer, just knowing that she’s [CRC worker] there and that she’s helping me get through it, you know? Because like I said, normally I would go to two appointments and I’m on the run. (CRC Client 5)

Interviewees also spoke of the value of CRC workers assisting clients to develop a range of skills to build their confidence in living in the community, including support with learning how to use smartphones and computers, public transport, and in obtaining identification such as birth certificates.

##### Support with health and substance use

Clients emphasised CRC’s role in addressing complex mental health, substance use and trauma. CRC workers support clients through relational casework, and their role in facilitating access to GPs, psychologists, psychiatrists, and community mental health services and attending appointments to ensure clients receive prescribed medications, was frequently highlighted:

You’ve got to make sure that they are linked in with community mental health…. most of them are on medication… and they haven’t been released with medication. Sometimes you have to find a doctor and sit in a doctor’s [office]. (CRC Staff 7)

For clients on antipsychotic medications, post-release continuity of care is critical. Clients highlighted the emotional support provided by workers in managing PTSD and anxiety around service access.

Because of my PTSD I have issues. I get very confused and huge anxiety levels… Keeping on my medications is another thing. Also keeping up with my health.…any recovering addict or someone who’s had enough or don’t care about themselves, they give up on themselves because they don’t think anyone cares. (CRC Client 3)

Many participants highlighted the significance of dental care in improving both health and self-esteem around appearance. For some, longstanding dental pain was a driver of substance use:

When I got out, I had a big chip missing in my front teeth. It killed my confidence… [CRC worker helped to get] that chipped tooth fixed… it made me feel great… I also had my back teeth ripped out because they were infected and causing me a massive pain… that was a lot of the reasons I was using heroin just at the time, for the pain. (CRC Client 14)

The CRC AOD program used a harm minimisation approach that prioritised safe use practices and reduced risks of relapse or overdose. Workers were reported to help ensure clients had pharmacotherapy plans in place before release, addressing logistical barriers like daily clinic visits. Interviewees explained that CRC’s non-judgemental ethos fostered trust and openness between clients and workers.

##### Community connection and integration

Many clients described that in previous exits from prison, they had rarely received any formal supports as they had been disconnected or estranged from friends, family and the community. CRC’s ‘wrap-around’ model provided supportive referrals to other services, and helped clients access peer-led support, healing programs, victim support, and the NDIS. Staff frequently accompanied clients to initial appointments to ease anxieties which reportedly helped to ensure a smooth transition and comfort in accessing services independently.

Beyond clinical and practical needs, CRC staff emphasised the importance of fostering a sense of belonging and identity outside of the criminal justice system. Employment and education became priorities for clients as they stabilised, with CRC supporting connections to educational services such as TAFE.

Interviewees also identified workers facilitating safe social connections beyond the client-worker relationship, addressing the isolation clients can face post-release. Establishing healthy relationships was particularly complex for clients seeking to distance themselves from peers involved in substance use or the criminal justice system. To this end, participants noted the importance of support to identify patterns and heathy/unhealthy behaviours in relationships. Reconnecting with family, especially children, was identified as a key goal for many clients. CRC’s support in this area often involved navigating complex family dynamics, legal restrictions, and emotional challenges:

I hadn’t seen [daughter] in a couple of years… [CRC] gave me the emotional support that I needed. And the courage to actually call [child welfare authorities]… but they supported me, and I ended up seeing her, and now I go there every weekend. (CRC Client 11).

##### Flexible, outreach, relational and long-term support

The method of delivering support was recognised as crucial to CRC’s effectiveness in reducing contact with the criminal justice system. CRC’s flexible outreach model allowed workers to meet clients at locations and times that suited their needs to improve feelings of safety and comfort, particularly for those experiencing trauma-related mental health concerns. This flexibility significantly enhanced client engagement, particularly during the period immediately following release from prison. Many clients, however, exited custody into unstable or chaotic circumstances, making it difficult to adhere to rigid appointment schedules or travel to distant offices.

We’re focusing on people that really would have fallen through the gaps if we weren’t here… most [people] will not want to go and will be very resistant to going to an office… [due to] mental health issues, anxiety about groups… (CRC Staff 5).

The pre-release and throughcare components of the service delivery model were highlighted as pivotal to establishing early rapport and facilitating smoother transitions post-release.

##### Respectful, non-judgemental, strengths-based, compassionate and consistent support

Participants consistently emphasised the quality and style of CRC workers’ approach as critical. A core strength of CRC’s model was its non-judgemental and relational ethos that supported honest dialogue and genuine trust between workers and clients. This approach allowed clients to reflect on their substance use or past behaviours without fear of stigma, enabling opportunities for reflection and growth:

I had a lapse about a month ago, month and a half ago. They [CRC] were the first persons that I reached out… I didn’t feel guilty or anything like that and without them I think I would be back in addictions now… there was no judgment… wasn’t like most organisations you get that will put that sort of stigma on you. (CRC Client 4).

Regular, consistent contact was highlighted as crucial for building and maintaining trust. Workers proactive check-ins helped alleviate clients’ feelings of isolation and reinforced their sense of self-worth. This ongoing support, combined with genuine care and respect, had a positive impact on client’s confidence and willingness to seek help:

They’ve made me feel like it’s okay to ask, they’ve given me the confidence back to actually ask for help. (CRC Client 3)

Many clients also valued workers who shared lived experiences of the criminal justice system or substance use, which facilitated a deeper sense of connection and understanding:

They understand because they’ve been there and done it too… If I break down on the phone… they understand why I’m doing it… They’ve actually made me feel that it’s okay to say I’m having a shit day. (CRC Client 3).

This personal connection often made the relationship feel less clinical and more authentic, enabling clients to engage in ways they might not with other mainstream AOD services. Participants identified that they viewed their primary relationship as being with individual workers rather than with CRC as an organisation, though the organisational culture was seen as reinforcing these positive relationships. Many described CRC workers as a counterbalance to the dehumanising effects of institutionalisation, and felt a sense of loyalty, belonging, and family with CRC staff.

The confidentiality of CRC’s services further bolstered trust, particularly for clients wary of disclosing drug use due to potential repercussions from parole or other authorities. This harm-minimisation approach encouraged open discussions about substance use, and ways to use safely, as well as to minimise use or access other help if needed.

They only want the best for you. They don’t push you into anything you don’t want to do… It’s only at your time, at your pace. (CRC Client 4)

Clients often measured progress not only by external achievements but also by internal growth and stability:

Through CRC, I know that I am [on the right track]. They tell me that I am because they see the difference – they seen me when I first got out of jail to what I am now…even if I can’t, they can see it. (CRC Client 4)

In summary, interviewees highlighted that the effectiveness of CRC’s model lies not only in its focus on addressing the practical needs of people leaving prison with histories of complex disadvantage, but also the way in which this support is delivered: flexible outreach that is relational, non-judgemental, strengths-based, and tailored to the needs of the person.

## Discussion

Despite government commitments to reducing reoffending in Australia, the incarceration and reincarceration rates of people with complex needs continues to rise (McCausland & Baldry, 2023). Studies have shown that there is lack of services and knowledge around finding effective support for people leaving custody (Doyle et al., 2022; Treloar et al., 2021), and that this can contribute to cumulative and entrenched disadvantage for people experiencing poverty, disability, and cycles of imprisonment (Baldry et al., 2015; McCausland & Baldry, 2017). In NSW, over 8,393 people were discharged from prison on expired sentences or parole in 2023 (NSW Bureau of Crime Statistics and Research, 2024). CRC is able to provide reintegration support for only a small proportion of those people; this study indicates that its model of reintegration support could be adopted more broadly to support larger numbers of people and substantially reduce the social and economic costs of incarceration. Interrupted time-series analysis of court and custody outcomes found that CRC clients experienced a significant reduction in criminal justice system contact over the period studied, and this was corroborated in a comparison analysis with a similar cohort of people with complex needs who did not receive CRC support. These findings make an important contribution to the expanding evidence base of quasi-experimental studies examining throughcare and reintegration programs that contributed to improvements in participant outcomes and reductions in their contact with the criminal justice system (Grella & Rodriguez, 2011; Griffiths et al., 2017; Miller & Miller, 2015, 2015; Visher et al., 2017), showing that reintegration support can be an effective means of reducing reoffending and reincarceration rates including for people with complex needs. This study further indicated that the CRC model is cost-effective, with estimated cost savings amounting to millions of dollars in criminal justice-related costs for each new annual cohort.

The qualitative components of this study revealed the profound and persistent disadvantage faced by people enmeshed in the criminal justice system, with substance use and imprisonment emerging as intertwined and predictable outcomes of systemic inequities. The qualitative data also revealed systemic deficiencies within correctional healthcare that directly undermine the health of people leaving prison and their reintegration into the community. Interview respondents frequently spoke of people being released from prison without medication and the difficulties in accessing prescriptions in the community, unless supported by a program like CRC. These accounts not only reflect the consequences of fragmented prison healthcare but also highlight the way that community-based organisations are required to fill these gaps. While the social determinants of incarceration are well-documented (McCausland & Baldry, 2023), what has been underexplored is *how* service delivery models can effectively respond to these entrenched structural issues. Many programs emphasise individual change, yet the qualitative findings suggest that effective support requires acknowledging not only clients’ personal experiences and unmet needs but also the broader systems that funnel them into the criminal justice system. Service systems had regularly failed CRC clients interviewed for this study, but CRC’s person-centred and flexible approach helped to connect them with relevant services and programs to enable effective reintegration. CRC clients noted that the flexible, outreach, person-centred, proactive, relational non-judgemental and non-exclusionary support and advocacy offered by CRC workers was life-changing in terms of their substance use, recidivism, and a broad range of health and social outcomes. The findings echo previous research that it is important to maintain personalised programs for people leaving prison (Borzycki & Baldry, 2003), and that pre-release engagement, a housing first approach, and community based programs are important factors for effective supports to people leaving prison (Sotiri, 2016). The systematic review of qualitative evaluations of throughcare programs referenced earlier concluded that interpersonal skills of case workers, access to social support and housing, and continuity of case worker relationships throughout the pre-release and post-release period were key social and structural factors in program success (Kendall et al., 2018); the findings of this study confirm this. The qualitative study findings also highlight that reintegration is not a linear process but a complex, multifaceted journey shaped by evolving relationships with substance use and incremental gains in stability and wellbeing.

This study demonstrates the value of taking a mixed methods approach that considers the efficacy of reintegration support on clients’ health, wellbeing and associated social and economic outcomes, informed by the expertise and voices of people who have experienced reintegration and those working intensively with them, as well as on their recidivism rates. The qualitative interviews highlighted that success for CRC clients extended beyond merely avoiding re-incarceration; for some, progress meant reduced or safer substance use while for others, it involved securing safe housing or achieving seemingly small but meaningful milestones, such as paying bills on time. Including client-centred methods like analysis of wellbeing survey data enabled the capturing of progress using clients’ own metrics of success throughout their time on the program. This data also provided important information including that clients’ self-care and relationships slightly deteriorated towards the end of the support period, suggesting that there are difficulties involved in support cessation. The qualitative interviews gave an in-depth picture of the practical and relational kinds of support that people with complex needs require within the context of structural and systemic disadvantage that can significantly reduce their contact with the criminal justice system. While the client-centred studies weren’t able to capture the experiences of people after they had completed their programs with CRC, analysis of former clients’ court and custody outcomes was possible through administrative data, including in comparison to an appropriate cohort with complex needs. This enabled the tracking of the ongoing impact of CRC’s model of support including the savings to the criminal justice system.

The participants in this evaluation were individuals with complex support needs transitioning from prison to the community in NSW, Australia. While the specificity of this group may limit applicability to broader populations receiving generalised community-based AOD support, the challenges faced by people leaving prison as highlighted in this study – including systemic and structural barriers – are representative of those encountered across Australian states and territories (Borzycki & Baldry, 2003; Doyle et al., 2022; Kirwan et al., 2019; Treloar et al., 2021). The CRC programs evaluated included structured components that are common amongst reintegration models elsewhere in Australia, such as case management, a housing-first approach, and AOD support, which increases the transferability of these findings to other settings using similar approaches to post-release support.

The CRC programs evaluated were implemented within the distinct legislative, policy, and community-based service context of NSW. While criminal justice and most health services (including AOD) are state-governed – which shapes how the findings of this study may translate across jurisdictions – as noted, many of the challenges faced by people leaving prison are shared across Australian states and territories. Importantly, there is significant variation *within* NSW itself. The CRC programs under evaluation were not only delivered in metropolitan regions but also in regional and remote areas, and participants in this evaluation reflected this geographic spread. This enhances the relevance of the findings across other parts of Australia, though differences in local cultural, policy and service contexts should be considered when applying the results of this evaluation elsewhere.

### Limitations

For quantitative analysis, interrupted time series analysis is a quasi-experimental design that helps to identify an intervention effect when a randomised trial is not possible (or ethical). The analysis enabled measurement of changes over time before and after a point of intervention (in this case the commencement of the first CRC AOD or transition program participation). However, it is possible that other things may explain these changes. Because the date of intervention varied in this study, a major common event was unlikely to explain the results but individual factors cannot be ruled out.

The addition of a comparison analysis helps to control for the effects of changes over time due to things other than engagement with CRC. However, there were some underlying differences between the CRC cohort and the comparison group, notably a predominantly male sample in the comparison group while the CRC cohort had more of a gender balance. Women experience slightly lower re-offending rates in this analysis and according to official statistics for New South Wales (Pisani, 2022). Therefore, these differences were controlled for in the analysis. A further limitation related to the comparative interrupted time series analysis is that the historical contact with courts and custody by CRC clients in this study was not known in detail in advance. After the initial matching of the CRC client group with the cohort from the MHDCD Databank (based on prison exit and AOD usage), it became apparent that the CRC clients had histories of more intensive contact with the criminal justice system prior to engaging with CRC. The comparative interrupted time series method controlled for these differences in baseline trends and observable characteristics; however, further research assessing the effectiveness of CRC-like programs would benefit from a two-step approach where the samples could be selected from a larger comparison pool and matched based on offending and imprisonment histories, as well as other characteristics and indicators of compounded disadvantage.

The small comparison pool and differences between the groups, meant that statistical matching techniques, such as propensity score matching, were not feasible in this study. Finding matches would be difficult and result in a very small matched sample, unlikely to be representative of CRC clients. Matching on key characteristics (i.e. exit from prison in the same period and AOD issues) and controlling for other observed differences enabled us to conduct comparative interrupted time series modelling to strengthen the findings from the interrupted time series alone, for the subset of CRC clients who were referred from custody. However, due to residual confounding from unobserved differences (such as access to mainstream services) CRC program effects may be underestimated by the comparative interrupted time series models.

For the qualitative study, the primary limitation is that those clients who were willing and able to consent and subsequently participate in the interview had likely gained a reasonable level of stability in their transition into the community, and therefore might report more favourable outcomes than other clients. In discussing recruitment of clients with CRC staff, workers did note that some of their clients would find it too difficult to sit through an hour-long interview for various reasons or might not be able to consent due to the influence of substances. The evaluation relied on the qualitative data and thematic analysis in identifying the elements of CRC’s model that effectively supported people to break cycles of substance use, disadvantage and criminal justice contact, so the experiences of clients who were not willing or able to consent and participate may not be reflected. This kind of selection bias has been noted as a limitation across many other evaluations of programs supporting people transitioning from prison to communities (Kendall et al., 2018).

The cost benefit analysis was limited to short-term criminal justice outcomes which was the scope of the available quantitative data. However, the qualitative findings identified a wide range of social benefits to CRC clients some of which may incur short-term costs to services, but over time would reduce reliance on intensive-post release services, child protection and welfare services and associated costs. A future evaluation could measure cost-benefit more broadly by including a longer follow-up period and linking to a range of health and human services data.

## Conclusions

This study has demonstrated that an intensive, flexible, outreach model of support that meets the basic welfare, housing and health needs of people with complex needs exiting prison can significantly reduce their contact with the criminal justice system and produce economic benefits for government. Through a mixed methods approach, the evaluation showed that CRC’s programs are associated with a significant reduction in reoffending and time spent in custody, as well as contributing many important health, wellbeing and social outcomes.

## Data Availability

The data analysed in this study are confidential and cannot be shared.

## Footnotes

1 An additional sub-study that is not presented in this paper due to space constraints involved compilation of two case studies of CRC clients matched with individuals from a linked administrative databank which enabled comparative analysis of their pathways and social and economic outcomes (Sotiri et al., 2021).

2 The models adjust for the number of days available (exposure time) where a particular year is not fully observed. For example, due to the person turning 18 or dying during the year, or during the index year which was divided into days pre- and post-index date. For court outcomes (which were analysed based on the offence date) and number of new custody episodes, the models also adjust for days in custody in the period which reduces the number of days available.

3 The index date for the intervention group, before and after which comparisons are made, was the exit date of the prison episode during which they were first referred to CRC. The index date for the comparison group was the date of the first prison exit in 2014–2017.

4 The court data in the databank were available until December 2017 but this was based on the date of the court finalisation. As the analysis was based on the date of offence, the data were restricted to offence dates up to the end of 2016 to avoid bias from omitted observations due to the lag time from date of offence to date of finalised court appearance.

5 For underlying output and calculations, see Section 6 of Sotiri et al. (2021).

## Abbreviations

AOD: Alcohol and Other Drugs
BOCSAR: Bureau of Crime Statistics and Research
CRC: Community Restorative Centre
MHDCD: Mental Health Disorders and Cognitive Disabilities
ROD: Reoffending Database
SURE: Substance Use Recovery Evaluator
